# Investigating Human Neurovascular Coupling Using Functional Neuroimaging: A Critical Review of Dynamic Models

**DOI:** 10.3389/fnins.2015.00467

**Published:** 2015-12-18

**Authors:** Clément Huneau, Habib Benali, Hugues Chabriat

**Affiliations:** ^1^Laboratoire d'Imagerie Biomédicale, UPMC Paris 06, Centre National de la Recherche Scientifique U7371, Institut National de la Santé et de la Recherche Médicale U1146, Sorbonne UniversitésParis, France; ^2^Institut National de la Santé et de la Recherche Médicale U1161, Université Paris Diderot, Sorbonne Paris CitéParis, France; ^3^AP-HP, Hôpital Lariboisière, Service de Neurologie and DHU NeuroVascParis, France

**Keywords:** cerebral blood flow, computational modeling, magnetic resonance imaging, microcirculation, hemodynamics

## Abstract

The mechanisms that link a transient neural activity to the corresponding increase of cerebral blood flow (CBF) are termed neurovascular coupling (NVC). They are possibly impaired at early stages of small vessel or neurodegenerative diseases. Investigation of NVC in humans has been made possible with the development of various neuroimaging techniques based on variations of local hemodynamics during neural activity. Specific dynamic models are currently used for interpreting these data that can include biophysical parameters related to NVC. After a brief review of the current knowledge about possible mechanisms acting in NVC we selected seven models with explicit integration of NVC found in the literature. All these models were described using the same procedure. We compared their physiological assumptions, mathematical formalism, and validation. In particular, we pointed out their strong differences in terms of complexity. Finally, we discussed their validity and their potential applications. These models may provide key information to investigate various aspects of NVC in human pathology.

## Neurovascular coupling in functional neuroimaging

### Functional neuroimaging and dynamic models

Synaptic activity and activation of neurons increase energy consumption by local neurons and astrocytes. This additional energy demand is mainly driven by activities of ion pumping (Attwell and Laughlin, 2001) and by various associated metabolic processes (Iadecola and Nedergaard, 2007). This energy is produced locally from glucose and oxygen supplied by blood through local small vessels. In response to transient neural activity nearby vessels dilate, substantially increasing CBF. The exact physiological function served by this large increase of flow remains unclear. This mechanism, termed functional hyperemia, was initially thought to be driven by an oxygen debt (Magistretti et al., 1999). This concept is now discarded in favor of complex mechanisms involving different vasoactive agents (Iadecola and Nedergaard, 2007; Attwell et al., 2010).

**Figure 1:**
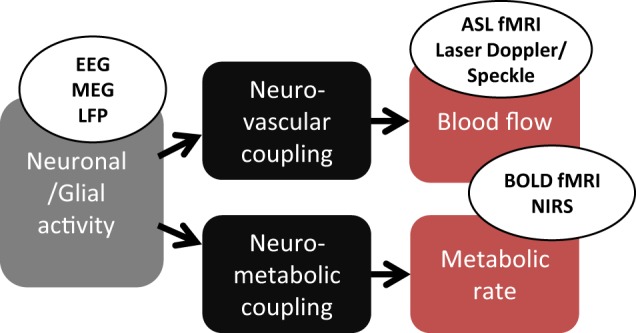
**Physiological mechanisms monitored in classical functional neuroimaging**. Neuronal/glial activity provokes, by a neurovascular coupling, an increase of blood flow. In parallel, a neurometabolic coupling increase the oxygen consumption. EEG, electroencephalogram; MEG, magnetoencephalogram; LFP, local field potential (intracerebral electrode); ASL, arterial spin labeling; BOLD, blood-oxygen-level dependent; NIRS, near infrared spectroscopy.

Neurovascular coupling (NVC) can be defined as the phenomenon that links a transient neural activity to the corresponding increase of CBF. The development of several functional neuroimaging techniques for evaluating *in vivo* cerebral functions is based, at least partially, on NVC. Measured in functional magnetic resonance imaging (fMRI), the blood oxygenation level-dependent (BOLD) contrast has become widely used to map brain activation in animals and humans in response to multiple types of neural stimulations (Ogawa et al., 1990). At the voxel level, the BOLD signal is related to changes of blood volume and deoxyhemoglobin concentration at a typical spatial resolution of 1 or 2 mm and temporal resolution of about 1 s (Buxton, 2013). Optical techniques, such as near-infrared spectroscopy (NIRS), can also measure blood oxygenation variations associated with transient neural activity. Conversely to fMRI, optical techniques have worse spatial resolution and are unable to explore deep brain structures but have a high temporal resolution that can reach 1 ms (Strangman et al., 2002). All these techniques are also noninvasive and can be used in humans. Despite this advantage, oxygen-based methods only perform indirect measurement of functional hyperemia (Figure 1). More comprehensive investigations of NVC would require additional information on hemodynamics. In animal models, optical methods as laser Doppler or laser Speckle flowmetry are currently used to measure blood velocity and flow changes within the superficial cortical layers at depth of about 500 μm (Fukuda et al., 1995). In humans, the development of fMRI led to other techniques that allow observation of purely hemodynamic phenomena not sensitive to oxygenation level. Mainly, arterial spin labeling (ASL) measures CBF variations but at a lower temporal resolution than using BOLD contrast (>2 s).

The different imaging techniques sensitive to cerebral hemodynamic changes are mostly used for inferring brain functions although the exact physiological mechanisms linking local blood flow and oxygenation changes to the corresponding focal variations of neuronal activity are still debated (Attwell et al., 2010; Hillman, 2014). Despite this imperfect knowledge, functional neuroimaging methods have been previously developed on the basis of more or less simple mathematical models of NVC and oxygen consumption (Friston and Dolan, 2010; Buxton, 2012). Particularly in fMRI, dynamic models are used to translate hemodynamic signal variations to neural activity, hence, resolving an inverse problem. Most of these models were primarily designed as descriptive models for depicting the transient hemodynamic and oxygenation changes in activated cerebral areas as the general linear model (Friston et al., 1995). However, some of them are constructed to mimic physiological mechanisms and offer the potential of estimating various biophysical parameters related to fMRI signal changes. Several are based on the hypothetical inflation or deflation of cerebral venules secondary to variations of intravascular pressure with inflow, as in the so-called Balloon model and Windkessel model (Buxton et al., 1998; Mandeville et al., 1999; Obata et al., 2004; Zheng and Mayhew, 2009). These models are essentially explanatory models as they are used to explain empirical observations in terms of theoretical underlying mechanisms. They usually include several compartments (Friston et al., 2000; Buxton et al., 2004; Huppert et al., 2007), each modeling a particular step in the global process from neural activity to observed signal (Figure 1). In the present review, we focus on compartments that specifically model NVC, in both descriptive and explanatory models developed for functional neuroimaging.

### Basis of neurovascular coupling

During neural activation, both neurons and astrocytes may act on arteriole smooth muscle cells (SMC) in response to glutamate release (Attwell et al., 2010). Neurons can play a direct vasomotor role through the delivery of nitric oxide (NO) and/or prostaglandin (PG), two potent vasodilators (Golanov and Reis, 1994; Li and Iadecola, 1994). Astrocytes can also dilate arterioles by acting on SMC through many intermediates such as PG, NO, epoxyeicosatrienoic acids (EET) or potassium release (Zonta et al., 2003; Takano et al., 2006). In addition, astrocytes are able to produce arachidonic acid (AA) that can act as a vasoconstrictor on the microvasculature (Metea and Newman, 2006) which suggests that NVC may not be exclusively related to dilating mechanisms. Finally, in addition to glutamate-mediated NVC, γ-Aminobutyric acid (GABA) was shown to have vasodilator effects on the microvasculature in cortical and subcortical regions but its exact involvement in NVC remains undetermined (Cauli et al., 2004; Kocharyan et al., 2007). Recent data support that neurons mainly contribute to the large and rapid vasodilation during NVC (Nizar et al., 2013; Lacroix et al., 2015). This mechanism of vasodilation is thought to start by neighbor arterioles and to back-propagate through endothelium-signaling along the vascular tree to reach larger arteries (Chen et al., 2014). Other data suggest a slower contribution of astrocytes on blood flow regulation that may be involved in the long-term functional hyperemia as well as in the basal flow control (Kim et al., 2015; Rosenegger et al., 2015). Traditionally, all these mechanisms are thought to occur at the level of SMC in the wall of arteries and arterioles considered as the unique location where local CBF is controlled. Nevertheless, recent results suggest that pericytes surrounding capillaries may also participate to vasodilation during brain activation (Peppiatt et al., 2006). Pericytes might be involved even faster than SMC in response to neural activity (Hall et al., 2014). However, a significant contribution of capillary pericytes to regional blood flow remains controversial (Hill et al., 2015). Their role may be restricted to a very local flow distribution between different capillaries. Thus, both arterioles and capillaries may participate in functional hyperemia but with different spatial scales.

Various pathological conditions, particularly those disrupting the neurovascular unit might alter the permanent adaptation of blood supply to local energy needs at the cerebral level. Accumulating evidence suggests that NVC is modified in cerebrovascular and degenerative disorders occurring in humans. In animal models of Alzheimer's disease, functional hyperemia was found impaired, as cerebrovascular autoregulation, long before the occurrence of amyloid plaques (Girouard and Iadecola, 2006). These dynamic changes were also detected in the absence of amyloid angiopathy and reproduced in normal mice with superfusion of amyloid-beta peptide (Aβ1-40) on the neocortex (Niwa et al., 2000; Park et al., 2004). In hypertensive mice, a decreased response to whisker stimulation was also detected after administration of angiotensin II (Kazama et al., 2003). Administration of losartan, an angiotensin II blocker, was recently found capable of rescuing the NVC in these models possibly through a reduction of oxidative stress and of superoxide products within the microvasculature (Ongali et al., 2014). In contrast, in APP mice, experimental data rather support that alterations of NVC may result essentially from neuronal and not from vascular dysfunction (Rancillac et al., 2012). In humans, a significant attenuation of cerebral blood flow (CBF) response to various stimulations was reported *in-vivo* before and after the occurrence of dementia in Alzheimer's disease (Hock et al., 1997). Modifications of flow reactivity after stimulations have been also reported in intracranial and extracranial vascular disorders (Hamzei et al., 2003) or after ischemic stroke, remote from the infarction related to transhemispheric diaschisis (Enager et al., 2004) and following various heterogeneous patterns (Krainik et al., 2005). Finally, functional or structural changes of arterioles or capillaries might also alter the adaptive response of the cerebral microvasculature. Dumas and coworkers recently reported significant changes of CBF response after visual stimulations in patients with probable cerebral amyloid angiopathy (Lin et al., 2011). Similar changes were detected in patients with sporadic hypertensive small vessel disease (Dumas et al., 2012). Modifications of pericytes surrounding capillaries or SMC in arterioles might be involved in these reactivity changes (Monet-Leprêtre et al., 2013).

**Figure 2:**
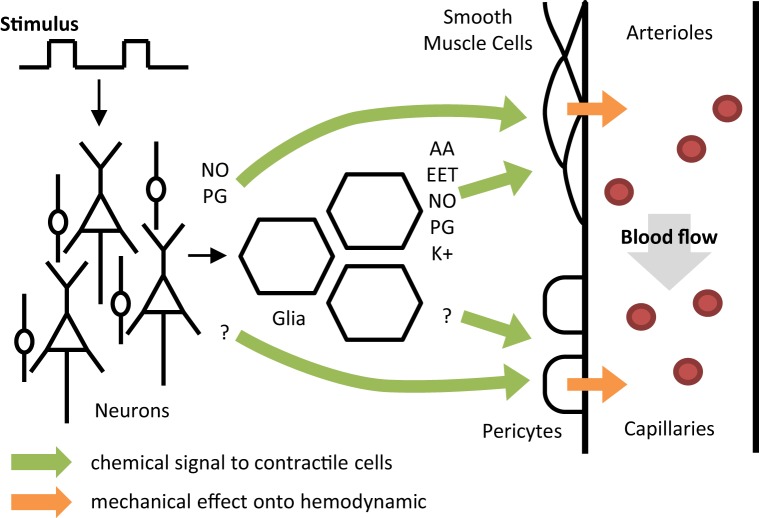
**A conceptual one direction model of neurovascular coupling**. Colored arrows represent the relationship between different activities: neurons, glia, smooth muscle cells, pericytes, and blood flow. NO, nitric oxide; PG, prostaglandin; AA, arachidonic acid; EET, epoxyeicosatrienoic acids; K, potassium.

In previous human studies obtained *in-vivo*, various alterations in flow response to specific stimulations were previously recorded (Donahue et al., 2012), but these neuroimaging observations remain difficult to interpret. The lack of further insight into the underlying NVC mechanisms is most likely related to methodological limitations such as the major restrictions in pharmacological manipulation studies in humans, the potential effects of medication used in pathological conditions and also to the lack of validated models to extract key physiological information from signal changes in functional imaging studies. At the microscopic scale, NVC is based on a complex system which is not fully understood (Figure 2). *In vivo* investigations in humans do not reach the microscopic resolution. In functional neuroimaging, a spatial unit (voxel) can include several dozens of arterioles and venules and hundreds of capillaries (Lorthois et al., 2011b). Therefore, modeling such data should be obtained at an intermediate level usually called the “mesoscopic scale” that will explore a large population of cells and vessels.

This review is aimed at reporting the most recent dynamic models of NVC used for interpreting functional neuroimaging results and discussing their validity relative to the current physiological knowledge. In order to link mesoscopic models with the current knowledge of NVC, we proposed to analyze these models based on a simplified representation of NVC (Figure 2) assuming that all mechanisms occur essentially in one direction but following different tracks from neural activity to functional hyperemia. The first step begins in neurons or glia and includes all mechanisms leading to changes observed in SMC or pericytes (four potential tracks). The second step mainly represents the effects of vessel changes on blood flow variations. Finally, the potential usefulness of these models for exploring NVC in cerebrovascular disorders is discussed from different perspectives.

## Analysis of dynamic models including NVC modeling

In this review, dynamic models were selected based on the following criteria: (1) the model should include modeling of an explicit relationship between the neural activity and local CBF changes; (2) the NVC modeling should correspond to the mesoscopic scale and simulate variations of blood flow observed at the fMRI voxel size; and (3) the model should contain less than tens of parameters. Among 403 articles detected in the English literature from 2000 to 2014 whose title contained the terms of “model” with those of “fmri,” “hemodynamic,” “blood flow,” or “(neuro) (electro)vascular,” we found 26 different models related to the hemodynamic response after a transient neural activity. Among them, we selected seven models that actually include some modeling of NVC. The other models were discarded for different reasons. A majority of models of the BOLD response focus on oxygen exchange and simply consider the flow similar to the neural activity or to the sensitive stimulus (Aubert and Costalat, 2002; Sotero and Trujillo-Barreto, 2007). Other authors proposed to model the propagation of blood pressure waves in the microvasculature and focus on the relationships between flow and volume in capillaries and veins (Kong et al., 2004; Drysdale et al., 2010; Aquino et al., 2012). However, these models did not provide any original approach of the control of arteriolar flow by the neural activity. Finally, we also discarded very detailed physiological models which were not relevant for neuroimaging data in human (Bennett et al., 2008). The seven selected models are listed in Table 1.

**Table 1:** **The seven dynamic models of neurovascular coupling selected from the literature**.

**Model name**	**Publications**	**Context**
Friston flow (FF)	Friston et al., 2000	Compartment of the Balloon model
Buxton flow (BF)	Miller et al., 2001; Buxton et al., 2004	Neural and NVC compartments of the Balloon model
Arteriolar compliance (AC)	Behzadi and Liu, 2005	Compartment of the Balloon model
Local electro-vascular coupling (LEVC)	Riera et al., 2006, 2007	Neural and NVC compartments of the Balloon model
3 compartments Windkessel (3CW)	Huppert et al., 2007; Mesquita et al., 2009	Compartment of a Windkessel model
Dilation-constriction (D-C)	Zheng et al., 2010	Independent model
Proximal integration (PI)	Kim et al., 2013; Kim and Ress, 2016	Compartment of a modeling of BOLD signal

Each of these models was then reviewed following the same procedure. We first addressed the central question behind its construction and the underlying biophysical mechanisms considered to model the NVC. Hence, we described each model regarding the NVC scheme in Figure 2. Then, we analyzed the mathematical formalism used for the most important parts of each model and described whether they were considered as linear or not. Although linearity may concern many aspects of modeling, herein, we chose to focus on the linearity of the relationships between the input and output amplitudes of models, a controversial aspect in the modeling of NVC mechanisms. We also proposed to classify the main parts of each mathematical model under review either as descriptive or explanatory. For this purpose, one part of a model was considered as purely descriptive when it only attempts to reproduce data using mathematical functions and without any hypothesis concerning the underlying physiological mechanisms. Conversely, it was considered as explanatory when the modeling was based on realistic underlying mechanisms, possibly using biophysical parameters. Models can include both descriptive and explanatory parts. Finally the validation obtained for each model was described, regarding the type of measures, their use in animal experiments or human studies and the number of individuals.

### Friston flow model

Friston and coworkers proposed in 2000 a model of NVC for analysis of fMRI data (Friston et al., 2000). Their initial objective was to complete the hemodynamic and energetic compartments of the Balloon model previously developed by Buxton (Buxton et al., 1998) that did not include NVC modeling, for improving the prediction of neural activity from the BOLD signal.

In the Friston Flow (FF) model, a flow-inducing chemical signal presumably controls blood flow variations at the arteriolar level. Hence, in this model, the arteriolar activity results directly from the input stimulus, assumed to be a good estimate of the neural response in activated regions. The chemical signal also depends on a blood flow feedback which simulates an auto-regulation process. A second order ordinary differential equation (ODE) is used to describe CBF variations resulting from the input stimulus. It is presented as a simple linear explanatory model using three parameters (Table 2): neural efficacy and feedback regulation gain that are mainly scaling factors and time decay for the chemical signal inducing arteriolar dilation.

**Table 2:** **Main aspects of complexity and validation in the selected models**.

**Model name**	**NVC compartment parameters**		**Validation data**		
	**Biophysical**	**Scaling**	**Input**	**Output**	**Specie**
FF	Chemical signal decay	Neural efficacy Feedback gain	Stimulus waveform	BOLD	Human
BF	–	Amplitude Duration Delay	Predicted neural activity	ASL	Human
AC	Baseline muscle arteriolar compliance Baseline arteriolar radius Maximum arteriolar radius Intravascular pressure Flow exponent Baseline arteriolar wall thickness Baseline passive stress fraction	Neural efficacy Decay constant Feedback gain	Stimulus waveform	BOLD	Human
LEVC	Neuronal energetic factors (x2) Flow-inducing signal susceptibility (x2) NO diffusion low-pass filter (x3) NO signal decay	Delay Feedback gain Scaling factors (x2)	EEG	BOLD	Human
3CW	Baseline arterial resistance Baseline capillary resistance Grubb's exponent Windkessel exponent Flow exponent Vascular transit time	Amplitude Duration Delay	LFP multi-unit activity	Laser Speckle	Rat
D-C	–	Delay Dilation factors (x4) Constriction factors (x4)	Current source density	Laser Doppler	Rat
PI	–	Flow change amplitude Damping time Flow oscillation frequency	Stimulus waveform	BOLD	Human

These parameters were estimated in BOLD fMRI experiments in four healthy individuals (Friston et al., 2000; Friston, 2002). No explicit comparison of output signal was reported so far. For validating the time decay, the only biophysical parameter included in the model, the authors found the estimated value to match the half-life of NO (between 0.1 and 1 s) but not that of K^+^ (about 5 s; Friston et al., 2000). Hence, this model hypothesizes that NO is the only vasoactive agent involved in NVC, contrary to what is now recognized in physiology (Attwell et al., 2010).

### Buxton flow model

After developing their initial model of venous inflated balloon (Buxton et al., 1998), Buxton and coworkers proposed a model that connects a neural compartment to the initial Balloon model through both a neurovascular and a neurometabolic coupling (Buxton et al., 2004). The Buxton flow (BF) model was proposed mainly to complete the physiological path from the stimulus to the BOLD response by taking into account experimental results such as the initial dip (Yacoub and Hu, 2001) and temporal nonlinearity of both flow and BOLD signals (Birn et al., 2001; Miller et al., 2001).

The BF model is purely descriptive at the level of NVC. Mathematically, this model consists in a linear convolution of the neural activity (typically the local field potential—LFP) with a flow response function. By definition this model makes no other assumption than the global linearity of NVC. The response function is a gamma-variate function, involving three parameters (Table 2): the normalized amplitude, duration and order (shape parameter) of the CBF impulse response (formalism equivalent to an ODE). For the neural compartment, Buxton and coworkers proposed a simple neural adaptation model linking excitatory and inhibitory activity after an input stimulus of the subject. This neural compartment can handle temporal nonlinearity between the stimulus and CBF response (Miller et al., 2001). For taking into account the possibility of a post-stimulus neural undershoot, a threshold constraint was added to the neural compartment.

Finally, no real data were provided in the 2004 article to validate this NVC model that includes a nonlinear neural adaptation. Nevertheless, previous experimental results (ASL fMRI in human) were consistent with the hypothesis that temporal nonlinearities are handled by the stimulus to neural activity rather that NVC (Miller et al., 2001). This supports the hypothesis of temporal linearity of the NVC compartment, although it does not validate the shape of the CBF impulse response used in the model.

### Arteriolar compliance model

After experimental data repeatedly showed that CBF variations and BOLD responses were modulated by the baseline level of CBF (Matsuura et al., 2000; Kemna and Posse, 2001), Behzadi and Liu proposed to include into the balloon model a specific compartment of NVC based on the arteriolar compliance (AC) for fitting experimental observations (Behzadi and Liu, 2005).

The AC model considers a flow-inducing chemical signal identical to that proposed in the FF model. Its originality is based on a specific modeling of the arteriolar wall compliance before generating the resulting blood flow (Davis and Gore, 1989; Lash et al., 1991). In this paradigm, arterioles have two distinct compliance components: an active compliance corresponding to SMC activities within the microvasculature and a passive compliance mainly corresponding to the non-contractile components of the vascular wall including the endothelium. At rest, SMC constrain the arterioles thus offering a low compliance to the flowing blood. During neural activation, SMC are forced to relax, increasing AC in order to increase blood flow. Once the arteriolar diameter reaches a threshold value (corresponding to the largest expansion of the endothelium wall), SMC are considered as totally relaxed while arterioles are constrained only by passive compliance. Mathematically, the authors used the linear ODE formalism of Friston and coworkers in their model (Friston et al., 2000) and added a nonlinear model based on mechanics formalism and the Poiseuille equation (Fung, 1997). Overall, the model is essentially explanatory and includes various biophysical parameters such as the baseline muscle/total compliance and the normalized maximum dilation radius of arterioles (Table 2).

This model was evaluated using BOLD experiments in healthy humans (Cohen et al., 2002; Behzadi and Liu, 2005). The baseline signal was found to increase linearly with expired CO2 from hypocapnic to hypercapnic levels. Conversely, the magnitude of the BOLD response to visual stimulation was found to decrease linearly with CO2. The (AC) model seems therefore capable of fitting the BOLD signal under variable conditions. This fitting is made possible by tuning, within constrained physiological range, baseline parameters as the resting state CBF or vascular wall compliance.

### Local electro-vascular coupling model

Riera and coworkers developed recently a framework combining EEG and fMRI signals in the setting of NVC modeling (Riera et al., 2006, 2007). They proposed a mesoscopic model that connects the initial neural stimulus to BOLD signal through a local electro-vascular coupling (LEVC) formalism. The activity of cortex layer V was specifically modeled using a neural mass approach with electrical dynamics considered as membrane potentials originating from pyramidal cells, feed-forward interneurons or feedback interneurons (Tagamets and Horwitz, 1998). This electrical part of the model that allows simulating realistic EEG signal was then coupled to the classical Balloon compartment (Buxton et al., 1998) for generating BOLD observations.

In this LEVC model, the authors assumed that NVC is mainly mediated through NO signaling from neurons to SMC. The NO calculation includes two steps corresponding to the synthesis and diffusion of NO. Extra-cellular NO concentration is first calculated based on a nonlinear weighted contribution of membrane potentials from the three neuronal populations. Diffusion of NO to SMC of nearby arterioles is thereafter modeled by linear low pass filtering. Finally, CBF is calculated using free NO concentration and a differential equation proposed by Friston et al. (2000). The LEVC model relies on 12 parameters for calculating CBF from membrane potentials (Table 2).

This model was validated by obtaining a nonlinear correlation between the stimulation frequency and the hemodynamic response. Indeed, the simulation showed a kind of resonance frequency producing the highest response which corroborated experimental observations in the rat barrel cortex (Hewson-Stoate et al., 2005) or in the human visual cortex (Singh et al., 2003). In a companion paper, Riera and coworkers applied their model to EEG and fMRI data obtained in two subjects (Riera et al., 2007). A numerical method was used for estimating several physiological parameters integrated to the neural mass and NVC parts of the model. These parameter estimations were not validated yet using physiological measurements. However, Rosa and coworkers have recently compared different variations of this model for integration of the neural input (Rosa et al., 2011). The results obtained support the choice of using the synaptic activity to control the flow during slow neuronal activity, but also argue to consider the neuronal firing rate for faster activity.

### 3-Compartments windkessel model

Huppert et al. extended the initial Windkessel model (Mandeville et al., 1999) to a 3 compartments model (3CW; Huppert et al., 2007). The 3CW model includes multiple compartments separating arteries, capillaries and veins for calculating the flow response. The model was further improved when the relevance of a linear transfer function between the neural and arteriolar changes was specifically investigated (Mesquita et al., 2009).

In the 3CW model, the NVC is obtained following two steps. The first compartment is mainly descriptive and based on linear functions from the neural activity to the arteriolar diameter. The second compartment is more explanatory and models the cascade influence of the arteriolar flow onto the capillary and venous flow according to a vascular passive inflation mechanism (so-called “Windkessel mechanism”). In the first part of the model, the authors designed a linear convolution with a gamma-variate impulse response function using three free parameters (amplitude, onset time, and width) to calculate the arteriolar diameter change related to the input stimulus (Table 2). In the second part, the arteriolar resistance changes are derived from vessel diameter variations and give access to the flow between the arteriolar and capillary compartments. Like many models derived from the Windkessel model, this model is based on electrical circuits formalism for calculating the hemodynamics in brain microvessels. In the 3CW model, the calculation is based on the hypothesis of a constant pial arterial pressure, capillary resistance and compliance, which was challenged by recent results (Peppiatt et al., 2006; Hall et al., 2014).

The 3CW model was validated using laser Doppler flowmetry (LDF) and local field potentials or multi-unit activities recorded in rats (Mesquita et al., 2009). Parameters of the flow impulse response were estimated for nine amplitudes of the same stimulus. Model outputs were finally found well correlated to experimental data but the nonlinearity pointed out using variable stimulus amplitudes was not found reproducible. Since this model calculates blood flow in three compartments, it may be of particular interest for discriminating and estimating functional alterations of arterioles, capillaries, or veins.

### Dilation-constriction model

The simple dilation-constriction (D-C) model has been initially proposed by Zheng et al. (2010) for taking into account the release of a vasoconstrictor agent that was recently observed during brain activation (Metea and Newman, 2006; Filosa and Blanco, 2007; Devor et al., 2008). Thus, this model integrates the conflicting roles of various vasoactive substances that can be responsible for dilation (NO, EET, PG, K^+^) or constriction (AA) of microvessels (Attwell et al., 2010). Both of these mechanisms that can occur simultaneously during brain activation are considered in parallel for modeling NVC.

The D-C model integrates two linear third order dynamic equations, one for dilation and the other for constriction of the microvasculature. Each part includes four parameters for setting the dynamics and efficacy of both effects. CBF is simply calculated with summation of both responses with a certain delay. Finally, the D-C model consists of two independent descriptive models for distinct physiological pathways but including parameters that have no biophysical meaning.

The D-C model was first tested using variations of current source density and LDF during electrical stimulation of rat whisker pad (Zheng et al., 2010). Optimization of parameters was performed to match experimental data. Parameters of the model were thereafter validated using a stimulation paradigm distinct from that initially used. The model provides excellent fitting of experimental CBF measures obtained in rats using Laser Doppler. The D-C model can also predict CBF changes in different stimulation paradigms based on fixed parameters. Thus, experimental validation obtained in this model further supports the hypothesis of concurrent dilation and constriction during neural activity (Metea and Newman, 2006).

### Proximal integration model

Kim and coworkers recently proposed a new model based on a mechanism called proximal integration (PI; Kim et al., 2013), similar to the emerging concept back-propagating vasodilation from arterioles to larger arteries. In the PI model, neural activity is assumed to generate a flow-inducing command that first reaches the nearby capillaries and that propagates later to larger vessels (arterioles then arteries) through astrocytes end-feet or pericytes (Itoh and Suzuki, 2012). Kim and coworkers later integrated this mechanism in a global model proposed to predict the oxygen time course following a brief neural activation (Ress et al., 2009).

In the PI model, any neural activation is considered to modify pericytes in the microvasculature and cause immediate dilation of local capillaries. The vasodilation signal then back-propagates through vascular branches to penetrating and pial arteries. Mathematically, the model used the electrical formalism for representing the blood flow resistance and compliance in the whole microcirculatory circuit. The neural activity is assumed to decrease suddenly the capillary resistance, hence leading the associated circuit to behave as a damped oscillator. This mechanism is actually translated as a convolution of the input stimulus signal with a damped oscillatory impulse response. The model requires three parameters which are not related to biophysical values: the amplitude, damping time and oscillation frequency.

To validate the corresponding overall model, outputs were compared with tissue-oxygen measurements in the visual cortex obtained from experiments in cats (Kim et al., 2013). A manual-automatic procedure was performed to estimate the PI model parameters but no fitting with flow measures for complete validation has been reported. Very recently, the same team included the PI model (now termed arterial impulse model) in a global modeling of the BOLD signal (Kim and Ress, 2016), very different from the so-called Balloon model. They exhibit better fits with BOLD signal than the Balloon model. While encouraging results, still no direct fits of the “pure” flow response are shown.

## Potential and limitations of selected dynamic models

The seven mesoscopic models of NVC reported in the literature largely vary on different aspects. All models were based on the most current knowledge of biophysical mechanisms presumably involved in NVC. However, the understanding of functional hyperemia has evolved considerably since the initial model reported by Friston and coworker. Thus, the central role of a primary NO signaling from neurons to arteriolar SMC in the FF model or in the LEVC model (Friston et al., 2000; Riera et al., 2006) is now challenged by the discovery of more complex mechanisms involving new players such as astrocytes or pericytes (Metea and Newman, 2006; Schummers et al., 2008; Hall et al., 2014). The most recent models such as the dilation-constriction (D-C) and PI models were built taking into account, at least partially, these new elements (Zheng et al., 2010; Kim et al., 2013). The mathematical construction also largely differs between these models. Some of them, the FF, BF, D-C, and PI models, are mainly descriptive and only attempt to replicate, using mathematical equations, the signal variations detected experimentally. However, despite this intrinsic limitation, these models may remain extremely useful for quantitative comparison between different subjects and populations. In contrast, the AC, LEVC, and 3 compartments Windkessel (3CW) models are more explanatory and integrate biophysical parameters that may be used for exploring various underlying biological processes. In the D-C model, the parameter estimation may help to discriminate alterations of mechanisms involved in dilation from those related to the constriction of the microvasculature. In the AC model as well as in the 3CW model, the Poiseuille equation used for describing the relationships between the arteriolar activity and the variations of CBF can provide detailed information about the post-stimulus flow response in pathological conditions. Although these methods are promising for investigating NVC in cerebrovascular disorders, explanatory models present a higher complexity than descriptive models, need a greater number of parameters and can include nonlinear operations. All these aspects will make the estimation of model parameters more difficult.

### Identifiability

Dynamic models can be used to reproduce real data with simulations and to identify a specific parameter value that allows the model output to fit real data. The identifiability of a model, corresponding to its capacity to estimate all parameters with the smallest variance, is a key property that was not systematically assessed. Deneux and Faugeras evaluated the identifiability of two models derived from the Balloon model including the FF model for modeling the NVC compartment (Deneux and Faugeras, 2006). The sensitivity of model output to variations of free-parameters was tested in this archetypal model. The results showed that large variations of different parameters have almost no effect on the output. The estimation of model parameters from observed data is then largely compromised since many parameter values will reproduce identical data. Hu and Shi pointed out also that the parameter identifiability in fMRI models largely depends on the complexity of temporal dynamics of model inputs and therefore is strongly related to the stimulation paradigm (Hu and Shi, 2010).

### Validity

All models have been previously validated, i.e., they were shown capable to fit *in vivo* data and to provide fitted values of their biophysical parameters corresponding to values obtained with other methods and already reported in the literature. This validation was made using different types of data. Human data obtained from BOLD contrast were used for validation of the FF, AC, LEVC, and PI models (Friston et al., 2000; Behzadi and Liu, 2005; Riera et al., 2007; Kim and Ress, 2016). The balloon model which plays a central part in most of these models is now largely questioned since it is mainly based on a passive venous expansion mechanism that is thought now to play a negligible role (Lorthois et al., 2011a). Fitting fMRI measures for investigating NVC would then require additional modeling using the most recent assumptions concerning hemodynamic changes or oxygen consumption mechanisms. The increased global complexity with a higher number of parameters can however reduce the global identifiability of the final model. Finally, two models, the 3CW and D-C models were validated at the individual level using direct measures with laser speckle or laser Doppler measures of local CBF in rats (Mesquita et al., 2009; Zheng et al., 2010). Excellent fits with real data were obtained providing an external validation not obtained in the other models. In addition, Zheng and coworkers used different paradigms for estimating parameters in the D-C model (Zheng et al., 2010), however they did not report any confidence interval of their estimates. In the PI model, most convincing validation was obtained fitting BOLD signal measured in human (Kim and Ress, 2016), as described above, but local blood flow signal was not considered although it appears essential for exploring NVC.

### Potential use

**Figure 3:**
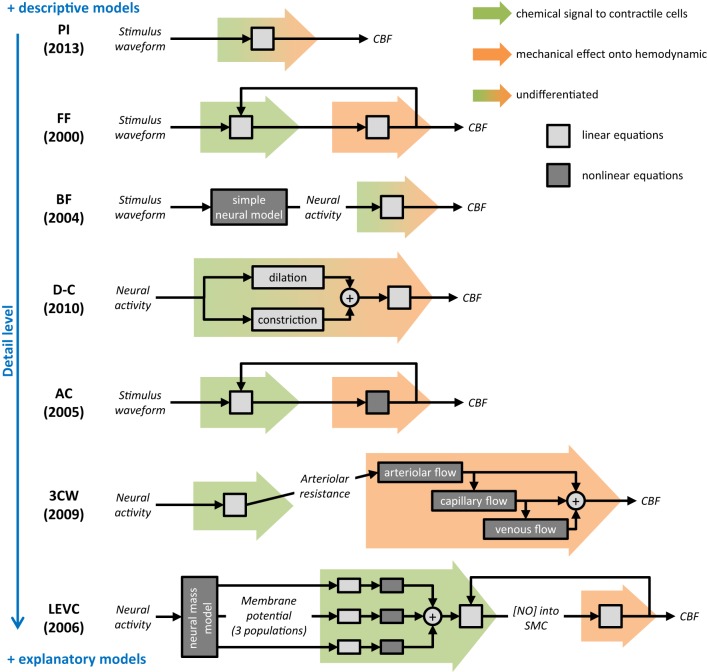
**Summary and comparison of selected dynamic models of neurovascular coupling**. As for the rest of this review, only the neurovascular coupling compartment is considered. For each model, a schematic represents the mathematical process proposed to calculate the output cerebral blood flow (CBF) from the input (stimulus waveform or neural activity). Background colored arrows refer to the two steps of NVC described in Figure 2: chemical signal from neurons/glia to contractile cells (green) and mechanical effect onto hemodynamic (orange). Boxes indicate either underlying equations are linear (light gray) or not (dark gray), and report the overall mathematical complexity. On the right, blue scale orders models regarding their detail level. Selected model names: PI, proximal integration; FF, Friston flow; BF, Buxton flow; D-C, dilation-constriction; AC, arteriolar compliance; 3CW, 3 compartments Windkessel; LEVC, local electro-vascular coupling.

Since the physiology of NVC is not yet fully understood, it remains difficult to determine which dynamic model is actually the best to capture a specific mechanism of NVC. The choice of a model largely depends on the experimental paradigm used for obtaining functional hyperemia as well as on the nature and resolution of measured data. Furthermore, models including a large number of unknown parameters (D-C and LEVC) are more difficult to interpret particularly using a single stimulation paradigm because of the limitation of identifiability. The choice of a model to investigate NVC also depends on the context and exact aim of the study. To compare NVC between different populations, descriptive models fitting the data (Figure 3) may be largely sufficient. The main advantage of descriptive models is that they do not include physiological parameters that can vary with the underlying pathological processes. Conversely, to infer on a specific underlying NVC mechanism, the choice of a model is particularly difficult when its validity is not fully established in humans and in normal physiological conditions. The use of multiple explanatory models with comparison of different results may be then useful for better interpretation. Finally, the results obtained using any model should be always interpreted with caution, not as facts but only for raising new hypotheses that will still require further validation.

## Conclusion

Different models including a specific NVC compartment were previously used in human and animal hemodynamic-based studies. However, these models were rarely built for specifically investigating NVC in normal or pathological conditions. While descriptive models appear potentially usable for comparing patients with cerebrovascular disorders from healthy subjects, explanatory models may offer new hypotheses for investigating *in vivo* some specific aspects of NVC in normal and pathological conditions. Before using these models for exploring NVC in healthy subjects and patients, the following aspects will need however to be considered; (1) the advantage and risk of using fixed parameter values obtained from the literature should be carefully evaluated according to the study aim and underlying pathological condition, (2) the number of variables needed and complexity of dynamic processes should be reduced to the minimum, (3) the linearity or nonlinearity of the underlying mechanisms should be carefully examined, and (4) the validity always discussed for both normal and pathological situations. We recommend that, in first approach, modeling of NVC in human should firstly restrict to the most macroscopic models (FF, BF, and PI; Figure 3). But these models may fail to fit certain dynamic of the blood flow response, for instance, occurring during longer activation or in pathological NVC. In these cases, the need of models with more parameters (D-C) or including nonlinearity may reveal relevant (AC). Finally, the most complex explicative models (3CW and LEVC) of NVC should be reserved to the modeling of microscopic flow signal imaging in animals, like two-photon imaging.

From our point of view, NVC has surprisingly been forsaken in modeling functional neuroimaging, especially in humans. This may be due to the poor understanding of the underlying physiological mechanisms, in addition to the focus of fMRI community on other aspects of the BOLD signal. Nowadays, there are growing issues to specifically investigate NVC in human pathologies such as neurodegenerative diseases. Recent progress in the understanding of NVC underlying mechanisms and development of new functional neuroimaging techniques (ASL or ultrafast ultrasound imaging) allow the development and validation of new and specific models of NVC. These models will be necessary to find new and noninvasive biomarkers of neurovascular diseases based on dynamic aspects of functional hyperemia.

### Conflict of interest statement

The authors declare that the research was conducted in the absence of any commercial or financial relationships that could be construed as a potential conflict of interest.
